# Influence of *APOE* genotype in primary age-related tauopathy

**DOI:** 10.1186/s40478-020-01095-1

**Published:** 2020-12-07

**Authors:** Andrew C. Robinson, Yvonne S. Davidson, Federico Roncaroli, James Minshull, Phillip Tinkler, Michael A. Horan, Antony Payton, Neil Pendleton, David M. A. Mann

**Affiliations:** 1 Division of Neuroscience and Experimental Psychology, Faculty of Biology, Medicine and Health, School of Biological Sciences, Salford Royal Hospital, The University of Manchester, Salford, M6 8HD UK; 2 Geoffrey Jefferson Brain Research Centre, Manchester Academic Health Science Centre (MAHSC), Manchester, UK; 3 Division of Informatics, Imaging and Data Sciences, Faculty of Biology, Medicine and Health, School of Health Sciences, The University of Manchester, Oxford Road, Manchester, M13 9PL UK

**Keywords:** Primary age-related tauopathy, Alzheimer’s disease, *APOE*, Cognition

## Abstract

The term “Primary age-related tauopathy” (PART) was coined in 2014 to describe the common neuropathological observation of neurofibrillary tangles without associated beta-amyloid (Aβ) pathology. It is possible for PART pathology to be present in both cognitively normal and cognitively impaired individuals. Genetically, Apolipoprotein E (*APOE*) ε4 has been shown to occur less commonly in PART than in Alzheimer’s disease (AD). Here, we investigate the relationships between PART, AD and those pathologically normal for age, with an emphasis on *APOE* and cognition, using 152 selected participants from The University of Manchester Longitudinal Study of Cognition in Normal Healthy Old Age and the Manchester arm of the Brains for Dementia Research cohort. *APOE* genotype differed between PART and AD with *APOE ε2* more common in the former and *APOE ε4* more common in the latter. Individuals with definite PART were less likely to be cognitively impaired than those with AD and those with pathology considered pathologically normal for age. We postulate that the lack of Aβ in definite PART cases may be due either to an increased frequency of *APOE* ε2 or decreased frequency of *APOE* ε4 as their resulting protein isoforms have differing binding properties in relation to Aβ. Similarly, an increased frequency of *APOE* ε2 or decreased frequency of *APOE* ε4 may lead to decreased levels of cognitive impairment, which raises questions regarding the impact of Aβ pathology on overall cognition in elderly subjects. We suggest that it may be possible to use the increased frequency of *APOE* ε2 in definite PART to assist neuropathological diagnosis.

## Introduction

For many years, neuropathologists have observed neurofibrillary tangles (NFTs) without associated beta-amyloid (Aβ) pathology in the brains of aged individuals both with and without cognitive impairment. NFTs are almost ubiquitous in the brains of older people [22]. Significant tau burden (Braak tau stage III-IV) but few Aβ plaques have been observed in 2–10% of individuals in community-based settings [15, 21, 29]. The inability to assign a diagnosis of Alzheimer’s disease (AD) to cognitively impaired individuals with these findings led to terms such as “senile dementia with tangles” or “tangle-only dementia” [11]. In those cognitively intact, “age-related changes” were often cited. To address this phenomenon, the term “primary age-related tauopathy” (PART) was suggested and consensus guidelines on diagnosis were proposed [6].

PART has been shown to be equally as common in males and females [8]. Although cognitive impairment can be present or absent in PART, the age group primarily affected (over 75 years) commonly report subjective memory issues. However, these memory complaints generally do not progress to dementia in those with a post-mortem diagnosis of PART [16].

PART is primarily diagnosed post-mortem. Macroscopically, no significant brain abnormalities are usually present although widespread cerebral atrophy has been documented in some cognitively impaired cases [1]. Microscopically, NFTs are found in similar regions to those affected by AD but with the notable exception of the neocortex. Thus, those with PART are generally considered Braak tau stage III or lower. For PART diagnosis, Aβ load should be minimal (or absent). A maximum Thal phase of 2 is required. Stratification into ‘possible PART’ and ‘definite PART’ is based on the level of Aβ burden (Table 1). Secondary pathologies have been noted in PART including cerebrovascular pathology [12], argyrophilic grains, hippocampal sclerosis, and TAR DNA-binding protein 43 (TDP-43) [13, 37]. Other secondary findings in PART, such as cerebral amyloid angiopathy (CAA) [12] and Lewy bodies [13] are less common. The presence of co-existing pathologies are unsurprising as mixed brain pathologies are often found in aged individuals [14].

**Table 1:** Inclusion criteria for the four groups used in the present study

Study group	Characteristics for inclusion
Normal pathology for age	Braak stage 0
	Thal phase 0–1
	No other significant pathological changes
AD pathological changes	Braak stage III or higher
	Thal phase 3 or higher
	No other significant pathological changes
Possible PART	Braak stage III or lower
	Thal phase 1–2
	No other disease associated with NFTs present
Definite PART	Braak stage III or lower
	Thal phase 0
	No other disease associated with NFTs present

It is well known that apolipoprotein E (*APOE*) ε4 carriers are at a greater risk of AD [32] whereas *APOE* ε2 carriers are thought to be at lower risk of AD [27] but at a greater risk of CAA–related haemorrhage [18]. Previous studies have shown that *APOE* ε4 is less frequently found in PART when compared to AD [2, 3, 6, 36]. Conversely, *APOE* ε2 has been shown to be more common in PART when compared to AD [3, 6]. Other studies have shown that microtubule associated protein tau (*MAPT*) *H1/H1* haplotype is more commonly found in PART than in AD [28].

Here, using selected participants from The University of Manchester Longitudinal Study of Cognition in Normal Healthy Old Age (UMLCHA) and the Manchester arm of the Brains for Dementia Research (BDR) cohort, we investigate the relationships between PART, AD and those pathologically normal for age, with an emphasis on *APOE* and cognition. We demonstrate that *APOE* genotype differs between PART and AD with *APOE ε2* more common in the former and *APOE ε4* more common in the latter. Furthermore, we show that individuals with definite PART were less likely to be cognitively impaired than those with AD and those with pathology considered pathologically normal for age implying that Aβ load may impact on cognitive outcome.

## Materials and methods

### Participants and study design

The present study uses selected participants from UMLCHA and the Manchester arm of the BDR cohort. Clinical and neuropathological characteristics of these cohorts have been previously described [24–26].

As the present study aims to assess relationships between AD, PART and those considered pathologically normal for age, there was a need to exclude a number of participants from each cohort (BDR 84 excluded; UMLCHA 54 excluded). The following exclusion criteria were applied to ensure suitability:

- Participants with primary neuropathological diagnosis other than AD, PART or pathologically normal for age
- Participants with significant confounding pathology (such as Lewy bodies). Note that cases with CAA were included in the analyses.
- Participants with no available *APOE* genotype
- Participants with a high probability of cognitive impairment due to vascular pathology (as measured by vascular cognitive impairment neuropathology guidelines [31]).

After applying the exclusion criteria, 152 participants (72 BDR and 80 UMLCHA) were considered eligible for the present study (Additional file 1: Table 1).

### Clinical assessment

The two cohorts conducted different assessment schedules to assign cognitive status. UMLCHA used the modified Telephone Instrument for Cognitive Status (TICSm) score with a cut-off point of 21 and BDR used the Clinical Dementia Rating (CDR) with a cut-off point of 0.5. Scores for TICSm and CDR have been shown to be highly correlated [30]. For the present study, we also used patient notes obtained via the participants’ general practitioner, cause of death (on death certification) and information gained from Brain Bank Coordinator (PT) to ensure the assignment of cognitive status at death was as accurate as possible.

### Pathological methods

The pathological methods associated with these cohorts have been previously described in detail [25, 26].

In short, donated brains were cut down the mid-line to result in two hemispheres. One hemisphere (usually the left) was fixed in 10% neutral buffered formalin for at least three weeks. The other hemisphere was frozen at −80 °C.

Standard blocks were dissected from fixed tissue (as according to BDR protocols) and processed into paraffin wax blocks. One paraffin section (6 µm) was cut from each block and stained with haematoxylin and eosin. Further paraffin sections (6 µm) were cut and used in immunohistochemistry for Aβ (Cambridge Bioscience, clone 4G8, 1:3000), tau proteins phosphorylated at Ser202 and Thr205 (Innogenetics, monocolonal AT8, 1:750), phosphorylated α-synuclein (polyclonal antibody (#1175), 1:1000 [23]), phosphorylated and non-phosphorylated TDP-43 (Proteintech, polyclonal antibody, 1:1000) and p62 (BD Transduction Labs, monoclonal, 1:100). For antigen retrieval, sections were either immersed in 70% formic acid for 20 min (for Aβ only) or microwaved in 0.1 M citrate buffer, pH 6.0 (all other antibodies) prior to incubation with primary antibody.

Consensus criteria were used to ascertain presence and stage of neurodegenerative disease. Final neuropathological diagnoses were assigned by experienced neuropathologists (DM & FR).

### Study group assignment

Criteria for inclusion into the four study groups included in this study can be found in Table 1.

### Genetic analysis

DNA was extracted from frozen brain tissue using REDExtract-N-Amp™ Tissue PCR Kit (Sigma) or from blood (3 cases). The *APOE* genotype was determined using routine polymerase chain reaction methods [34].

### Statistical analyses

Chi squared test was used to analyse whether there were differences in sex, severity of CAA, frequency of *APOE* ε4 allele(s) and frequency of *APOE* ε2 allele(s) between allocated pathology groups. Fisher’s Exact test was used when the expected count was less than five. *T* test assessed differences in age at death.

Logistic regression was used to investigate whether adjustment for sex and age at death made any difference to significant outcomes when analysing presence of *APOE* ε4 allele(s) and presence of *APOE* ε2 allele(s) between allocated pathology groups.

A *p* value of < 0.05 was considered significant.

## Results

### Demographics

Demographic information, stratified by pathology group, can be found in Table 2. Overall, 61.2% of participants were female. 50% of all participants were cognitively impaired. Mean age at death was 84.4 (± 9.2) years.

**Table 2:** Demography of eligible cases stratified by pathology group

	Normal for age		AD pathological changes		Possible PART		Definite PART	
	*n*	%	*n*	%	*n*	%	*n*	%
Sex (female)	9	60.0	49	59.0	23	79.3	12	48.0
Cognitive impairment at death	4	26.7	66	79.5	6	20.7	0	0
Age at death (Mean ± s.d)	79.7 (± 14.1)		83.0 (± 9.2)		88.3 (± 6.1)		87.1 (± 6.4)	

There was a significantly greater proportion of females in the possible PART group compared with the AD pathological changes group (χ^2^ = 3.848; *p* = 0.050) and the definite PART group (χ^2^ = 5.771; *p* = 0.016). As expected, there was a significantly greater proportion of cognitively impaired individuals in the AD pathological changes group when compared with the pathologically normal for age group (χ^2^ = 17.388; *p* < 0.001), the possible PART group (χ^2^ = 32.395; *p* < 0.001) and the definite PART group (χ^2^ = 51.119; *p* < 0.001). Likewise, there were significantly more cognitively impaired individuals in the possible PART group when compared with the definite PART group (χ^2^ = 5.819; *p* = 0.016). There were also significantly more cognitively impaired participants in the pathologically normal for age group when compared with the definite PART group (χ^2^ = 7.407; *p* = 0.006).

Mean age at death was significantly higher in the possible PART group compared with the pathologically normal for age group (*p* = 0.037) and the AD pathological changes group (*p* = 0.001). Similarly, mean age at death was significantly higher in the definite PART group compared with the AD pathological changes group (*p* = 0.015).

### CAA

In the AD pathological changes group, 45.8% of individuals had moderate to severe CAA pathology. In the PART pathology groups, moderate to severe CAA was much less prevalent (possible PART 20.7%; Definite PART 4%) and was completely absent from those considered pathologically normal for age. Proportionally, moderate to severe CAA was significantly more likely in with AD pathological changes than those with possible PART (χ^2^ = 5.674; *p* = 0.017) or definite PART (χ^2^ = 14.539; *p* < 0.001). There were no differences in severity of CAA between possible and definite PART groups (χ^2^ = 3.315; *p* = 0.069).

### *APOE* genotype

**Fig. 1:**
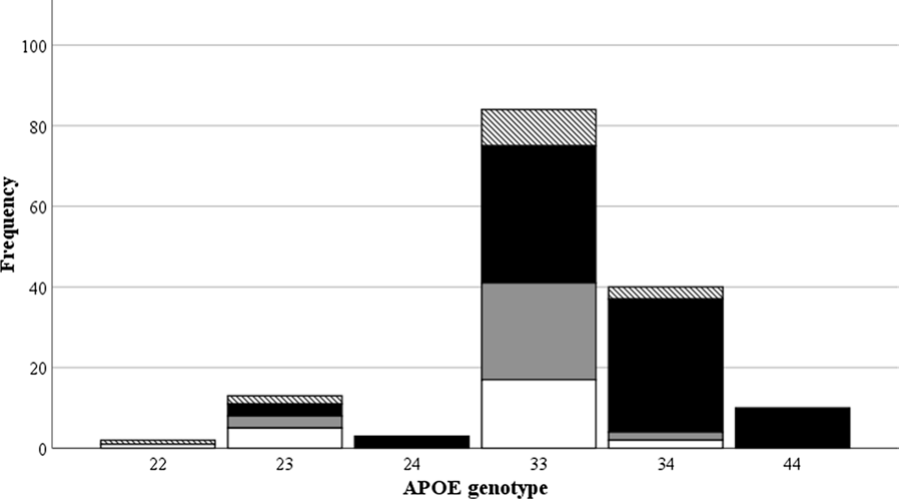
Breakdown of *APOE* genotype, stratified by pathology group (Hatched = Normal for age; Black = AD pathological changes; Grey = Possible PART; White = Definite PART)

Breakdown of *APOE* genotype can be found in Fig. 1.

As expected, *APOE* 3,3 was the most common genotype (55.3%). When stratifying by group, *APOE* 3,3 was the most common genotype in the pathologically normal for age (60.0%), possible PART (82.8%) and definite PART (68.0%) groups. All cases of *APOE* 4,4 were found in the AD pathological changes group. In addition, 39.8% of cases in the AD pathological changes group were *APOE* 3,4. The genotype *APOE* 2,4 was only present in the AD pathological changes group.

**Fig. 2:**
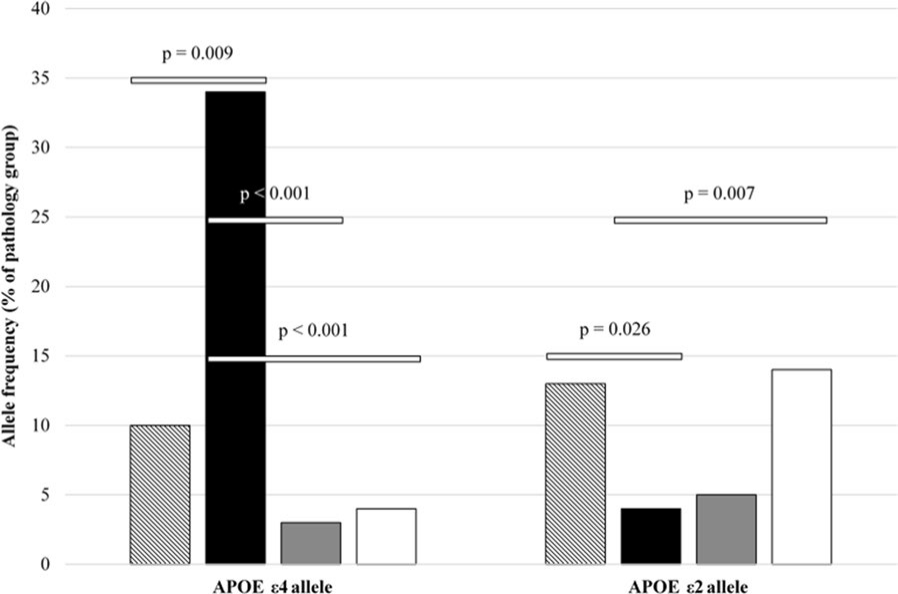
Allele frequency of *APOE ε*4/*APOE ε*2, stratified by pathology group (Hatched = Normal for age; Black = AD pathological changes; Grey = Possible PART; White = Definite PART)

Allele frequency of *APOE ε*4/*APOE ε*2, stratified by pathology group, can be found in Fig. 2.

The *APOE* ε4 allele appeared more frequently in the AD pathology group when compared with those pathologically normal for age (χ^2^ = 6.803; *p* = 0.009), those with possible PART (χ^2^ = 20.547; *p* < 0.001) and those with definite PART (χ^2^ = 17.297; *p* < 0.001).

*APOE* ε2 allele was found more frequently in the pathologically normal for age group when compared with the AD pathology group (χ^2^ = 4.957; *p* = 0.026). Likewise, *APOE* ε2 allele was more frequent in the definite PART group when compared with the AD pathology group (χ^2^ = 7.327; *p* = 0.007).

Regression analysis showed that sex and age at death had no effect on the outcome of significant results found for presence of *APOE* ε4 when comparing AD pathology group with pathologically normal for age group (OR = 5.928; *p* = 0.011), possible PART group (OR = 0.071; *p* = 0.001) and definite PART group (OR = 0.081; *p* = 0.001). Likewise, controlling for sex and age at death had no effect on the significant outcome found for presence of *APOE* ε2 when comparing definite PART group and AD pathology group (OR = 4.196; *p* = 0.032). However, sex and age at death affected the significant outcome found for presence of *APOE* ε2 when comparing pathologically normal for age group and AD pathology group (OR = 0.304; *p* = 0.113).

When considering CAA, the *APOE* ε4 allele was found more frequently in those individuals with moderate to severe CAA (χ^2^ = 17.778; *p* < 0.001) than none to mild CAA. There was no such difference when applying the same analysis to those with the *APOE* ε2 allele.

## Discussion

Our results show that the *APOE* ε2 allele appeared significantly more frequently in those found to be definite PART when compared with those who had AD pathological changes at death. Conversely, the *APOE* ε4 allele was more frequently found in those with AD pathological changes when compared with possible PART, definite PART or those pathologically normal for age. Present data are consistent with an earlier, less extensive study which pre-dates the inception of PART consensus criteria [9] and also a more recent study on individuals older than 85 years of age at death [3].

Previous studies have shown average age at death of those with PART to be higher than those with AD pathology [12]. Here, we show that, although those with AD pathological changes died at a younger age than those with PART changes, the difference was not statistically significant. This is probably due to different recruitment strategies of the two cohorts with BDR being case/control and UMLCHA more community or population based. These differences have been discussed in an earlier publication [26].

Cognitive impairment due to PART pathology is not yet fully understood. The majority of those with PART diagnosed post-mortem are considered to be non-demented [16] and it is thought that cognitive impairment is mainly associated with very severe PART pathology [6]. We show that cognitive impairment was only found in 20% of individuals with possible PART and was completely absent in those considered definite PART. Interestingly, there were significantly more individuals with cognitive impairment in the pathologically normal for age group when compared with those in the definite PART group. One interpretation could be statistical ‘noise’, though at *p* = 0.006, this would be unlikely. Another could be the inclusion of individuals at Thal phase 1, which reflects cortical Aβ pathology at its earliest stage, in the pathologically normal for age group as cognitive impairment can be apparent in individuals with a low burden of Aβ pathology [35]. This finding could also be due to the increased frequency of *APOE* ε2 in the definite PART group when compared with the pathologically normal for age group. It is worthy of note that our group has recently shown that carrying *APOE* ε2 may increase the chances of remaining cognitively normal [27].

The role of *APOE* ε4 as a risk factor for AD has been known for many years [4, 32] as has the role of *APOE* ε2 as a protective agent against AD [5]. *APOE* ε4 carriers are more likely to have significant Aβ load [20] whereas *APOE* ε2 carriers have a reduced Aβ burden [7]. Hence, in the present study, those with classic AD pathological changes (tau and Aβ) were more likely to carry *APOE* ε4 whereas those with definite PART were more likely to carry *APOE* ε2 and, thus, show no Aβ pathology.

This raises the question of what influence (if any) *APOE* ε2 has on the disease process. By definition, there is a lack of Aβ in PART and it is possible that *APOE* ε2 has an effect on amyloidogenesis or clearance of Aβ [17]. Likewise, it is also possible that the lack of Aβ in PART may be due to an absence of *APOE* ε4, which is known to strongly facilitate the deposition of Aβ [19], rather than the presence of *APOE* ε2, which may simply have a ‘neutral’ effect. However, a recent study [10] has shown that Aβ levels in those with *APOE* 2,4 genotype increased at half the rate with respect to increasing age when compared with those with *APOE* 3,4 which suggests that *APOE* ε2 has an effect on Aβ deposition even in those with a coexisting *APOE* ε4 allele. If *APOE* ε2 does in fact play a part, it may prevent deposition of Aβ by binding and stabilising it in brain parenchyma or vessels. If Aβ is excreted from the brain via perivascular channels and the role of *APOE* ε4 protein is to stabilise Aβ within the brain tissue and vessels, then the lack of Aβ in brain tissue in those with *APOE* ε2 ought to result in low levels of CAA. Here, we show that this is the case as those with *APOE* ε4 were more likely to have moderate to severe CAA whereas there was no such difference when applying the same analysis to those with the *APOE* ε2.

Another potential role for *APOE* could be the facilitation of NFTs in PART. Work using induced pluripotent stem cells which were differentiated into cultures of forebrain excitatory neurons has already shown that the presence of *APOE* ε4 can speed up the spread of tau pathology [33]. Another study has shown that the *APOE* ε2 allele is associated with a greater tau burden in brains with progressive supranuclear palsy (PSP) and that those with *APOE* ε2/ε2 genotype were found to have increased risk of PSP and corticobasal degeneration [38]. However, we have shown a lack of NFTs in those considered pathologically normal for age with *APOE* ε2 which would argue against such a role. Hence, the role of *APOE* ε2 seems to be restricted to any influence it may have over Aβ deposition. Although NFTs and Aβ coexist in AD, it is clear that Aβ is not (always) a prerequisite for NFT formation as, in PART, NFTs can occur independently of Aβ deposition. It is possible that, even in AD, the two pathological events are coincidental rather than causal.

## Conclusions

The differences in *APOE* allele frequency between the groups in the present study supports the conclusion that PART and AD are distinct entities. We show that those with definite PART are significantly more likely to carry *APOE* ε2 allele(s) and be considered cognitively normal at death compared to those with AD pathological changes. In addition, those with possible/definite PART are less likely to carry *APOE* ε4 allele(s) than those with AD pathological changes. We postulate that the lack of Aβ in definite PART cases may be due either to an increased frequency of *APOE* ε2 (or decreased frequency of *APOE* ε4) in these cases. Similarly, an increased frequency of *APOE* ε2 (or decreased frequency of *APOE* ε4) leads to decreased levels of cognitive impairment, which raises questions regarding the impact of Aβ pathology on overall cognition in elderly subjects. It may be possible to use the increased frequency of *APOE* ε2 in definite PART to assist neuropathological diagnosis.

## Supplementary information

**Additional file 1** .**Table 1**. Neuropathological overview of the 152 eligible participants. Description of data: Breakdown of neuropathological findings which was used to determine pathology groups for the present study. (XLSX 23 kb)

## Data Availability

The datasets used and/or analysed during the current study available from the corresponding author on reasonable request.

## Abbreviations

Aβ: beta-amyloid
AD: Alzheimer’s disease
APOE: Apolipoprotein E
BDR: Brains for Dementia Research
CAA: cerebral amyloid angiopathy
CDR: clinical dementia rating
MAPT: microtubule associated protein tau
NFTs: neurofibrillary tangles
PART: primary age-related tauopathy
PSP: progressive supranuclear palsy
TDP-43: TAR DNA-binding protein 43
TICSm: modified Telephone Instrument for Cognitive Status
UMLCHA: The University of Manchester Longitudinal Study of Cognition in Normal Healthy Old Age
